# The Dual Role of the 16mer Motif Within the 3′ Untranslated Region of the Variant Surface Glycoprotein of *Trypanosoma brucei*

**DOI:** 10.1111/mmi.70031

**Published:** 2025-11-17

**Authors:** Majeed Bakari‐Soale, Christopher Batram, Henriette Zimmermann, Nicola G. Jones, Markus Engstler

**Affiliations:** ^1^ Department of Cell and Developmental Biology, Biocentre University of Würzburg Würzburg Germany

**Keywords:** 16mer motif, antigenic variation, *Trypanosoma brucei*, VSG

## Abstract

The variant surface glycoprotein (VSG) of African trypanosomes is essential for the survival of bloodstream form parasites. These parasites undergo antigenic variation, an immune evasion strategy in which they periodically switch VSG expression from one isoform to another. The molecular processes central to the expression and regulation of the VSG are however not fully understood. In general, the regulation of gene expression in trypanosomes is largely post‐transcriptional. Regulatory sequences, mostly present in the 3′ UTRs, often serve as key elements in the modulation of the levels of individual mRNAs. In *T. brucei* VSG genes, a 16mer motif within the 3′ UTR has been shown to be essential for the stability of *VSG* transcripts and abundant VSG expression. This motif is 100% conserved in the 3′ UTRs of all transcribed and non‐transcribed VSG genes. As a stability‐associated sequence element, the absence of nucleotide substitutions in the 16mer is however exceptional. We therefore hypothesised that the motif is involved in other essential roles/processes besides the stability of the *VSG* transcripts. In this study, we demonstrate that the 100% conservation of the 16mer motif is not essential for cell viability or for the maintenance of functional VSG protein levels. We further show that the intact motif in the active VSG 3′ UTR is neither required to promote VSG silencing during switching nor is it needed during differentiation from bloodstream forms to procyclic forms. Ectopic overexpression of a second VSG, however, requires the intact 16mer motif within the ectopic VSG 3′ UTR to trigger silencing and exchange of the active VSG, suggesting a role for the motif in transcriptional VSG switching. The enigmatic 16mer motif therefore appears to play a dual role in transcriptional *VSG* switching and *VSG* transcript stability.

## Graphical abstract

A fully conserved 16mer motif is required in the silent VSG to trigger efficient silencing of the expression site (ES)‐resident VSG and a successful exchange of the VSG coat.

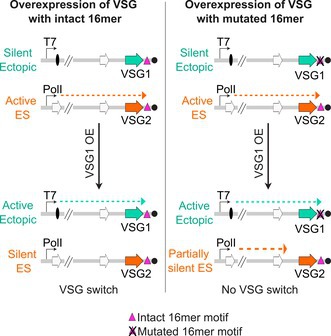

## 1 Introduction

The African trypanosome *Trypanosoma brucei* is a unicellular protist parasite of medical and veterinary importance. The parasite is strictly extracellular and is transmitted between its mammalian hosts by an insect vector, the tsetse fly. In the mammalian host, *T. brucei* thrives in the blood and tissue fluids despite being exposed to the host's immune system. Infection by the parasite can become chronic, lasting for months or years. The ability of the parasite to maintain chronic infections is due to constant changes in the identity of its surface coat through the process of antigenic variation (Vickerman 1978; reviewed in Horn 2014).

The *T. brucei* cell surface is covered by a dense layer of variant surface glycoprotein (VSG) composed of 10 million molecules linked to the cell surface via a glycophosphatidylinositol (GPI) anchor (Cross 1975; Grünfelder et al. 2002; Schwede and Carrington 2010). Each individual *T. brucei* cell expresses a single VSG out of a repertoire of over 2000 VSG isoforms (Cross et al. 2014). The actively expressed *VSG* occupies one of approximately 15 telomeric loci referred to as expression sites (ES) (Hertz‐Fowler et al. 2008; Hutchinson et al. 2016). Only a single ES associated with an extranucleolar RNA polymerase I (RNA pol‐I) transcription compartment, called the expression site body (ESB), is transcriptionally active in individual cells (Chaves et al. 1998; Navarro and Gull 2001). The active VSG sub‐compartment associates with a VSG exclusion (VEX) complex that mediates allelic exclusion, thus ensuring monoallelic VSG expression (Faria et al. 2019). The active VSG gene can however be exchanged with a silent VSG gene by either homologous recombination of a silent VSG into the active ES or transcriptional activation of a previously silent ES and silencing of the active ES (in situ switch) (Taylor and Rudenko 2006; Alsford et al. 2012; McCulloch et al. 2014). The mechanism of transcriptional VSG switching remains unclear.

VSG is the most abundant protein in the mammalian life cycle stages, comprising about 10% of the total protein (Wang et al. 2010). *VSG* mRNA is also the most abundant mRNA in blood stream trypanosomes with a half‐life reported between 1 and 4.5 h (Ehlers et al. 1987; Fadda et al. 2014; Ridewood et al. 2017). The high abundance of VSG protein and the very high stability of the *VSG* transcripts compared to other genes transcribed from the same ES has been attributed to RNA pol‐I transcription and post‐transcriptional control of VSG expression. *T. brucei* and related kinetoplastids have their genomes organised into long polycistronic transcription units. Most genes in these organisms are therefore constitutively transcribed into primary polycistronic transcripts, which are then processed into individual transcripts by a coupled *trans*‐splicing and polyadenylation reaction (Lebowitz et al. 1993). The regulation of gene expression in trypanosomes is therefore largely post‐transcriptional (Clayton 2019). The untranslated regions (UTRs) of mRNAs play critical roles in the post‐transcriptional regulation of gene expression. *Cis*‐regulatory elements in the 3′ UTR in particular have been shown to be involved in the regulation of mRNA stability and translation efficiency in trypanosomes (Berberof et al. 1995; Hotz et al. 1997; Erben et al. 2014; Ridewood et al. 2017; Jojic et al. 2018). Two highly conserved motifs, an 8mer and 16mer, exclusively present in the 3′ UTRs of VSGs have been implicated in the post‐transcriptional control of VSG expression. The 8mer motif is thought to be involved in a fail‐safe mechanism that ensures destabilisation of accidentally produced VSG transcripts in procyclics (do Nascimento et al. 2021). The 16mer motif on the other hand has been shown to be essential for *VSG* mRNA stability (Berberof et al. 1995; Ridewood et al. 2017). Two mechanisms describing the involvement of the 16mer motif in the maintenance of *VSG* mRNA stability have been proposed recently. Viegas et al. (2022) suggested that the 16mer is required for N6‐methyladenosine (m^6^A) modification of the poly(A) tails of *VSG* mRNAs. This m^6^A modification prevents deadenylation and promotes *VSG* mRNA stability. do Nascimento et al. (2021) on the other hand identified and described the RNA binding protein CFB2 as a 16mer binding factor mediating *VSG* mRNA stability by recruitment of a stabilising translation‐promoting complex.

The fact that the 16mer motif is 100% conserved across all *T. brucei* strains and isolates has so far gone unnoticed. Therefore, we asked why a simple mRNA‐stabilising motif of 16 nucleotides should not show a single polymorphism, even in silent, non‐transcribed copies of VSG genes. This is highly unusual. There is a stability‐associated 16 nucleotide sequence element in the 3′ UTR of procyclin transcripts, which encode the major insect stage cell surface protein of trypanosomes. This sequence motif is not as highly conserved (Hehl et al. 1994; Furger et al. 1997). Also, the mRNA motifs M23 and M24 in yeast and the miR‐381 and miR‐219 sequence motifs in humans have stability‐associated functions but do not show 100% conservation (Shalgi et al. 2005; Xie et al. 2005; Santa‐Maria et al. 2015; Long et al. 2019).

Therefore, we hypothesise that there might be additional functions responsible for the 100% preservation of the 16mer motif. This possibility was tested in an exclusion procedure. The 100% conservation of the motif was not required for the expression of functional levels of VSG protein and hence is dispensable for cell viability. We also showed that the intact motif in the ES‐resident VSG 3′ UTR is neither required for VSG silencing during switching nor is it needed during differentiation from bloodstream forms to procyclic forms. However, 100% conservation of the 16mer motif within the 3′ UTR of an ectopic VSG was essential for triggering efficient VSG silencing and coat exchange during a transcriptional VSG switch. This additional role of the motif in the process of VSG in situ switching may be the driving force for its absolute conservation in *T. brucei* VSGs.

## 2 Results

### 2.1 Full Conservation of the 16mer Motif Is Not Essential for Expression of Functional Levels of VSG Protein

An extensive mutation analysis of the *VSG121* 3′ UTR was performed by targeted deletion or substitution of nucleotides within regulatory motifs and truncations of the UTR to identify specific sequences or sequence lengths influencing the regulation of VSG expression. The mutants were stably integrated into the active expression site, upstream of the endogenous VSG221, to yield so‐called double‐expressor (DEX) cell lines (Figure S1A).

As anticipated, we found that the deletion of a segment from the 198 bp‐containing 3′ untranslated region (UTR) that includes either the complete 16mer motif (∆39–198), or specific portions of the 16mer (∆46–52 and ∆49–53), led to a significant reduction in VSG levels (see Figure S1A,B).

In contrast, inversion of the 8mer motif (Inv28‐35) or mutation of the region between the 8mer and 16mer motifs (AC41‐42TA) did not affect VSG expression levels.

However, we identified mutations of the 16mer motif (C61A and TGA46‐48ACT), which did not significantly impact VSG production (Figure S1A–C). Thus, in principle, the 100% conservation of the 16mer is not essential for parasite viability. This was confirmed by subsequent RNAi‐mediated depletion of the endogenous VSG221 in DEX‐parasites that featured a second VSG with a dysfunctional 16mer. Upon induction of RNAi these parasites revealed the typical lethal phenotype following pre‐cytokinesis arrest that has been described for VSG‐deficient trypanosomes. However, this detrimental phenotype was rescued if the 3′ UTR of the second VSG in the expression site contained one of the ‘tolerated’ mutations (Figure S2).

**FIGURE 1:**
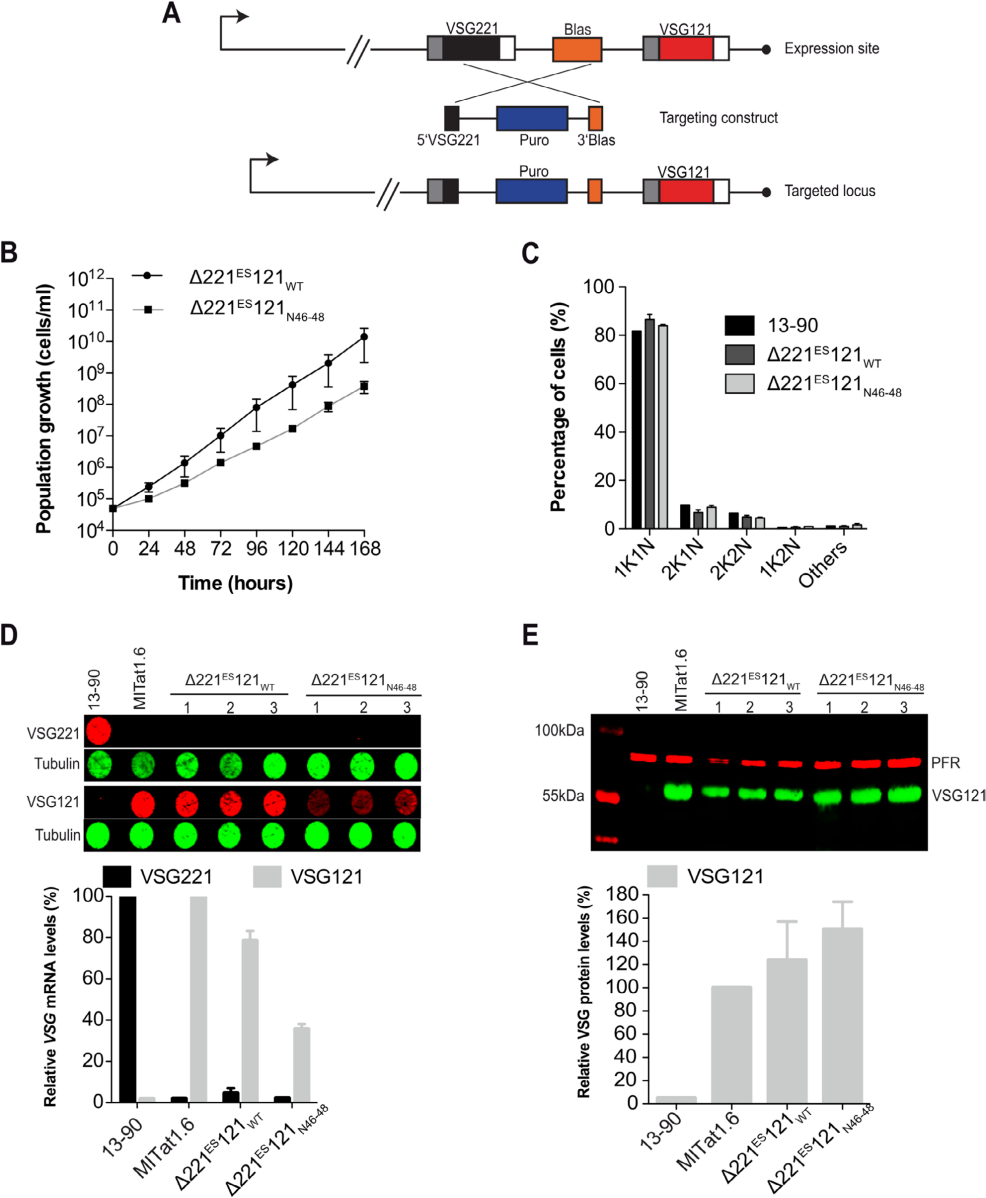
Substitution of the first three nucleotides of the 16mer motif supports expression of functional levels of VSG protein. (A) Schematic of the knockout strategy used in generating single‐expressor cells from double‐expressor cells with telomere proximal ectopic *VSG121*. The endogenous *VSG221* and blasticidin resistance (Blas) genes were replaced with a puromycin resistance gene (Puro). (B) Cumulative growth curve of single‐expressor cells expressing *VSG121* with wild‐type 16mer (∆221^ES^121_WT_) and mutated 16mer (∆221^ES^121_N4648_). Data are averages (mean) from three independent clones with error bars representing the standard error of the mean (SEM). (C) Cell cycle analysis of ∆221^ES^121_WT_ and ∆221^ES^121_N4648_ single‐expressor cell lines. The number of nuclei and kinetoplasts were analysed microscopically in 200 DAPI‐stained cells in each cell line. Three independent clonal cell lines were analysed, and data presented as mean percentages ± SEM. The parental 13–90 cell line was used as a control. No statistically significant differences were observed in any cell cycle stage between the 13–90 cells and the ∆221^ES^121_WT_ or ∆221^ES^121_N4648_ cells (prop. test, all *p* > 0.05; Bonferroni‐adjusted). (D) Quantification of *VSG* mRNA in ∆221^ES^121_WT_ and ∆221^ES^121_N46–48_ cells. *VSG221* and *VSG121* mRNA were quantified from RNA dot blots using fluorescently labelled probes normalised to *tubulin* mRNA (upper panel). *VSG221* and *VSG121* mRNA levels were expressed relative to levels in the parental 13–90 (VSG221) and MITat1.6 (VSG121) cells, respectively. Data are presented as mean ± standard error of the mean (SEM) of three independent clones. (E) Quantification of VSG protein in ∆221^ES^121_WT_ and ∆221^ES^121_N46–48_ cells. VSG121 protein levels were quantified from western blots and normalised to paraflagellar rod protein 1 and 2 (PFR) (upper panel). The levels of VSG121 protein were expressed relative to those found in the parental MITat1.6 cells. Data are presented as mean ± standard error of the mean (SEM) of three independent clones.

To confirm that parasites are viable with the tolerated mutation TGA46‐48ACT (also referred to as N46‐48) in the 16mer motif, we generated single‐expressor cells (∆221^ES^121_N46–48_) harbouring this mutation from stable DEX cells in which the ectopic VSG is downstream of the endogenous VSG (Figure 1A). DEX cells can be generated by inserting the second VSG either upstream or downstream of the endogenous VSG. Knock‐out of the endogenous *VSG221* resulted in parasites expressing only VSG121 from the VSG221 expression site. As a control, we generated a cell line with the wild‐type VSG121 3′ UTR (∆221^ES^121_WT_). Both cell lines were viable. The parasites expressing the VSG with a mutated 16mer motif (∆221^ES^121_N46–48_) exhibited a minor growth phenotype (Figure 1B). The ∆221^ES^121_WT_ cells had a population doubling time (PDT) of ~9 h while the ∆221^ES^121_N4648_ cells had a PDT of ~11 h. The slowed growth was not a result of cell cycle defects (Figure 1C). *VSG* mRNA was however 43% higher in the cells expressing a VSG with the wild‐type 16mer motif (79%) compared to the cells expressing a VSG with the mutated 16mer (36%). Both cell lines however had high VSG protein levels with ~36% higher levels in ∆221^ES^121_N4648_ cells (Figure 1D,E). The tolerated mutation appears to affect *VSG* mRNA abundance, which may impact the rate of VSG protein production resulting in the observed growth phenotype. In the steady state, however, the VSG protein abundance was high relative to the control in both the wild‐type and mutant. On the one hand this shows that trypanosomes can live with less than 50% of VSG mRNA. On the other hand, the experiment proves that the 100% conservation of the 16mer motif is neither required for cell viability in vitro nor for production of functional levels of the VSG protein.

### 2.2 An Intact 16mer Motif Is Not Required for Inclusion of N6‐Methyladenosine in Poly(A) Tails of *VSG* Transcripts

**FIGURE 2:**
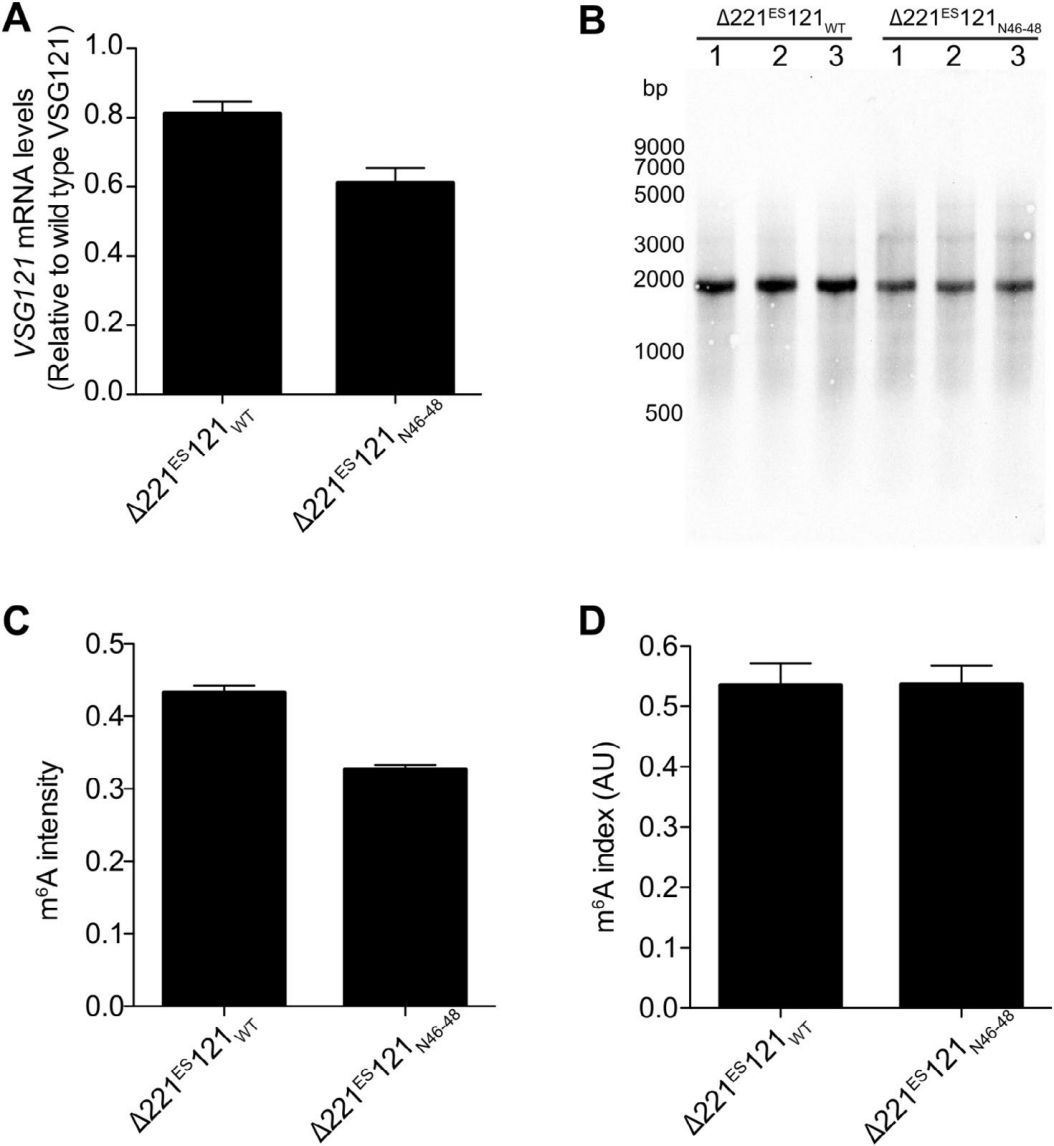
100% conservation of the 16mer is not required for m^6^A modification of the poly(A) tails of *VSG* mRNA. (A) Quantification of *VSG* mRNA in ∆221^ES^121_WT_ and ∆221^ES^121_N4648_ cells used in m^6^A immunoblots. *VSG121* mRNA was quantified from RNA dot blots, normalised to *tubulin* mRNA and expressed relative to the *VSG121* transcript levels in a MITat1.6 wild‐type cell line. Data are presented as the mean ± standard error of the mean (SEM) of three independent clones each. (B) Immunoblots showing m^6^A amounts in mRNA from ∆221^ES^121_WT_ and ∆221^ES^121_N46–48_ cells. (C) Intensity of the m^6^A signal in *VSG121* mRNA measured using ImageJ. The intensity of the m^6^A in the *VSG121* band was expressed relative to the intensity of the entire lane. Data are presented as the mean ± standard error of the mean (SEM) of three independent clones each. (D) m^6^A index in the two single‐expressor cell lines. The m^6^A index was computed as the ratio of the m^6^A intensity in (C) to the *VSG 121* mRNA levels in (A). Data are presented as the mean ± standard error of the mean (SEM) of three independent clones.

It has been suggested that the 16mer motif is required for N6‐methyladenosine (m^6^A) modification of *VSG* poly(A) tails contributing to the stabilisation of the *VSG* transcripts (Viegas et al. 2022). To test whether this process requires the full 16mer motif, we performed m^6^A immunoblotting of poly(A) + mRNA extracted from ∆221^ES^121_WT_ and ∆221^ES^121_N46–48_ cells. As a way of normalising the m^6^A signal to initial transcript levels, we computed an ‘m^6^A index’ as described (Viegas et al. 2022) by dividing the relative intensity of the m^6^A signal in *VSG* transcripts by the corresponding *VSG* mRNA levels measured by quantitative dot blots (Figure 2). Amounts of *VSG121* mRNA were 20% lower in the single‐expressor cells expressing *VSG121* with a mutated 16mer (∆221^ES^121_N4648_) compared to cells expressing *VSG121* with the wild‐type 16mer motif (∆221^ES^121_WT_) (Figure 2A). The immunoblot revealed m^6^A bands corresponding to the *VSG121* transcript size in both ∆221^ES^121_WT_ and ∆221^ES^121_N46–48_ cells with m^6^A intensities of 0.43 and 0.33, respectively (Figure 2B,C). The m^6^A index was however identical (~0.5 AU) in both ∆221^ES^121_WT_ and ∆221^ES^121_N46–48_ cells (Figure 2D). This suggests that 100% conservation of the 16mer motif is not required for m^6^A modification of *VSG* mRNA.

### 2.3 An Intact 16mer Motif Is Not Required for VSG Silencing During Differentiation From Bloodstream Form (BSF) to Procyclic Form (PCF)

**FIGURE 3:**
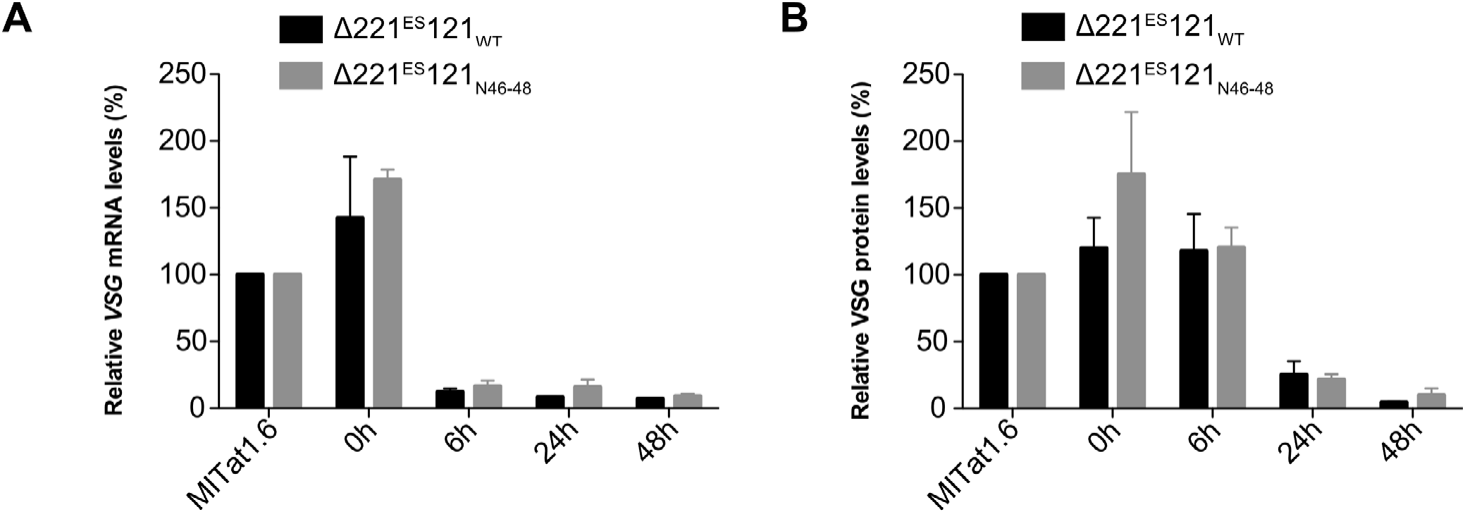
VSG silencing during differentiation from BSF to PCF occurs with similar kinetics in ∆221^ES^121_WT_ and ∆221^ES^121_N46–48_ cells. (A) *VSG121* mRNA levels in ∆221^ES^121_WT_ and ∆221^ES^121_N46–48_ cells during the course of differentiation. *VSG121* mRNA was quantified from RNA dot blots, normalised to *tubulin* mRNA and expressed relative to the *VSG121* transcript levels in a MITat1.6 wild‐type cell line. Data are presented as the mean ± standard error of the mean (SEM) of three independent clones, respectively. (B) VSG121 protein levels in ∆221^ES^121_WT_ and ∆221^ES^121_N46–48_ cells during the course of differentiation. VSG121 protein was quantified from protein dot blots, normalised to PFR and expressed relative to the VSG121 protein levels in a MITat1.6 wild‐type cell line. Data are presented as the mean ± standard error of the mean (SEM) of three independent clones, respectively.

Having established that the 100% conservation of the 16mer is neither essential for expression of functional levels of VSG nor for the m^6^A modification of *VSG* mRNA, we next investigated a potential role of the conserved motif in VSG silencing during differentiation from BSF to PCF. This differentiation process is one of two events where destabilisation/silencing of the active ES occurs. The process can be activated in vitro by cold‐shock and treatment with citrate and/or *cis*‐aconitate (Brun and Schönenberger 1981; Overath et al. 1986; Engstler and Boshart 2004). We performed the in vitro differentiation assay on ∆221^ES^121_WT_ and ∆221^ES^121_N46–48_ cells and monitored the *VSG121* mRNA and protein levels (Figure 3). Samples were collected 0, 6, 24 and 48 h after addition of 6 mM *cis*‐aconitate and incubation of parasites at 27°C. The *VSG121* mRNA and VSG121 protein levels were quantified using quantitative RNA and protein dot blots, respectively. A marked decrease of *VSG121* mRNA to less than 20% of the wild‐type levels was observed 6 h after induction of differentiation (Figure 3A). Less than 10% of *VSG121* transcripts were present by 48 h post‐induction. The decrease in mRNA was accompanied by a decrease in VSG protein to ~25% by 24 h (Figure 3B). These results are similar to reduced VSG amounts observed during differentiation in earlier studies (Ehlers et al. 1987). The kinetics of silencing of the *VSG121* mRNA and VSG121 protein were similar in both cells expressing *VSG121* with either a wild‐type or mutated 16mer motif, revealing that an intact 16mer motif is not essential for VSG silencing during differentiation. To directly investigate the effect of the 16mer mutation on a possible crosstalk between the procyclin EP1‐3′ UTR and the ES‐resident VSG 3′ UTR, we integrated EP1‐eYFP with a wild‐type EP1 3′ UTR into a transcriptionally silent rDNA spacer in ∆221^ES^121_WT_ and ∆221^ES^121_N46–48_ cells to generate the cell lines ∆221^ES^121_WT_EP1^Tet^ and ∆221^ES^121_N46–48_EP1^Tet^, respectively (Figure S3). Tetracycline‐induced overexpression of EP1‐eYFP had no significant impact on the ES‐resident VSG, irrespective of the *VSG121* harbouring either a mutated or the wild‐type 16mer motif. This observation suggests that a potential crosstalk between the EP1 and VSG 3´ UTRs is not dependent on an intact 16mer motif.

### 2.4 VSG Silencing During In Situ Switching Does Not Require an Intact 16mer in the Active VSG 3′ UTR

**FIGURE 4:**
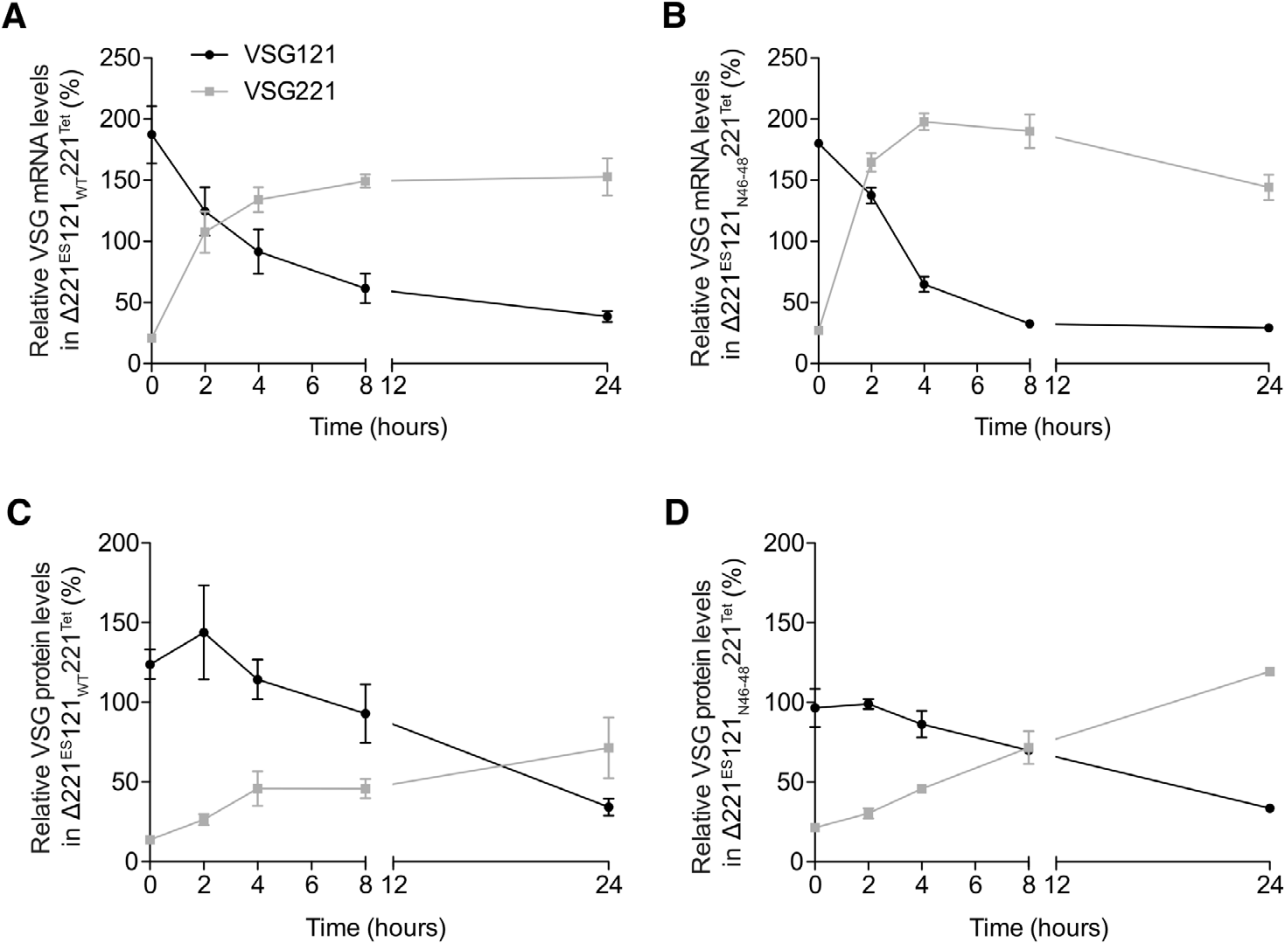
ES‐resident VSG121 is silenced with similar kinetics in both the wild‐type and mutated 16mer single‐expressor cell lines upon ectopic *VSG221* overexpression. *VSG* mRNA (A, B) and VSG protein (C, D) monitored in ∆221^ES^121_WT_221^Tet^ and ∆221^ES^121_N46–48_221^Tet^ cells during the course of *VSG221* overexpression. *VSG* mRNA and VSG protein levels were quantified from RNA and protein dot blots, respectively. The *VSG* mRNA was normalised to *tubulin* mRNA and the protein amounts normalised to PFR. The VSG expression levels are given relative to levels in the parental 13–90 (VSG221) or MITat1.6 (VSG121) cells and expressed as mean ± standard error of the mean (SEM). Data presented are from three independent clones for each cell line.

Apart from differentiation from BSF to PCF, VSG switching is the second event where silencing of the active VSG occurs in the parasite. Therefore, we next asked if the active VSG would have different silencing kinetics in the absence of the intact 16mer motif during switching. To test this possibility we used an inducible overexpression system that mimics transcriptional VSG switching (Batram et al. 2014) (Figure S4A). We integrated *VSG221* into a transcriptionally silent rDNA spacer in ∆221^ES^121_WT_ and ∆221^ES^121_N46–48_ cells. In the resulting ∆221^ES^121_WT_221^Tet^ and ∆221^ES^121_N46–48_221^Tet^ trypanosome lines, expression of the ectopic VSGs was driven by a tetracycline regulated T7 promoter. Upon induction of overexpression with tetracycline, the expression site resident *VSG121* transcript and protein levels decreased with similar kinetics in both the cell lines expressing *VSG121* with the wild‐type or with the mutated 16mer motif, respectively (Figure 4). Within 24 h of overexpression, the ectopic VSG221 became the most abundant VSG expressed, similar to what we described in our earlier observations (Batram et al. 2014; Zimmermann et al. 2017). We also observed continuous growth in both cell lines after induction with ∆221^ES^121_WT_221^Tet^ cells having a PDT of ~7.2 h compared to ~8.4 h for uninduced cells, whereas the induced ∆221^ES^121_N46–48_221^Tet^ cells grew slightly faster with PDT of ~7.1 h compared to ~7.7 h for uninduced cells (Figure S4B). Uninduced ∆221^ES^121_N46–48_221^Tet^ cells did not display a growth phenotype possibly due to leaky VSG221 expression as we observe about 20% VSG221 protein levels in uninduced cells (Figure 4). Transcriptional leakage has been reported in the vector used for the generation of these transgenic lines (Wirtz et al. 1999).

### 2.5 Ectopic Overexpression of a Second VSG Requires an Intact 16mer Motif to Trigger Efficient VSG Silencing and Coat Exchange

**FIGURE 5:**
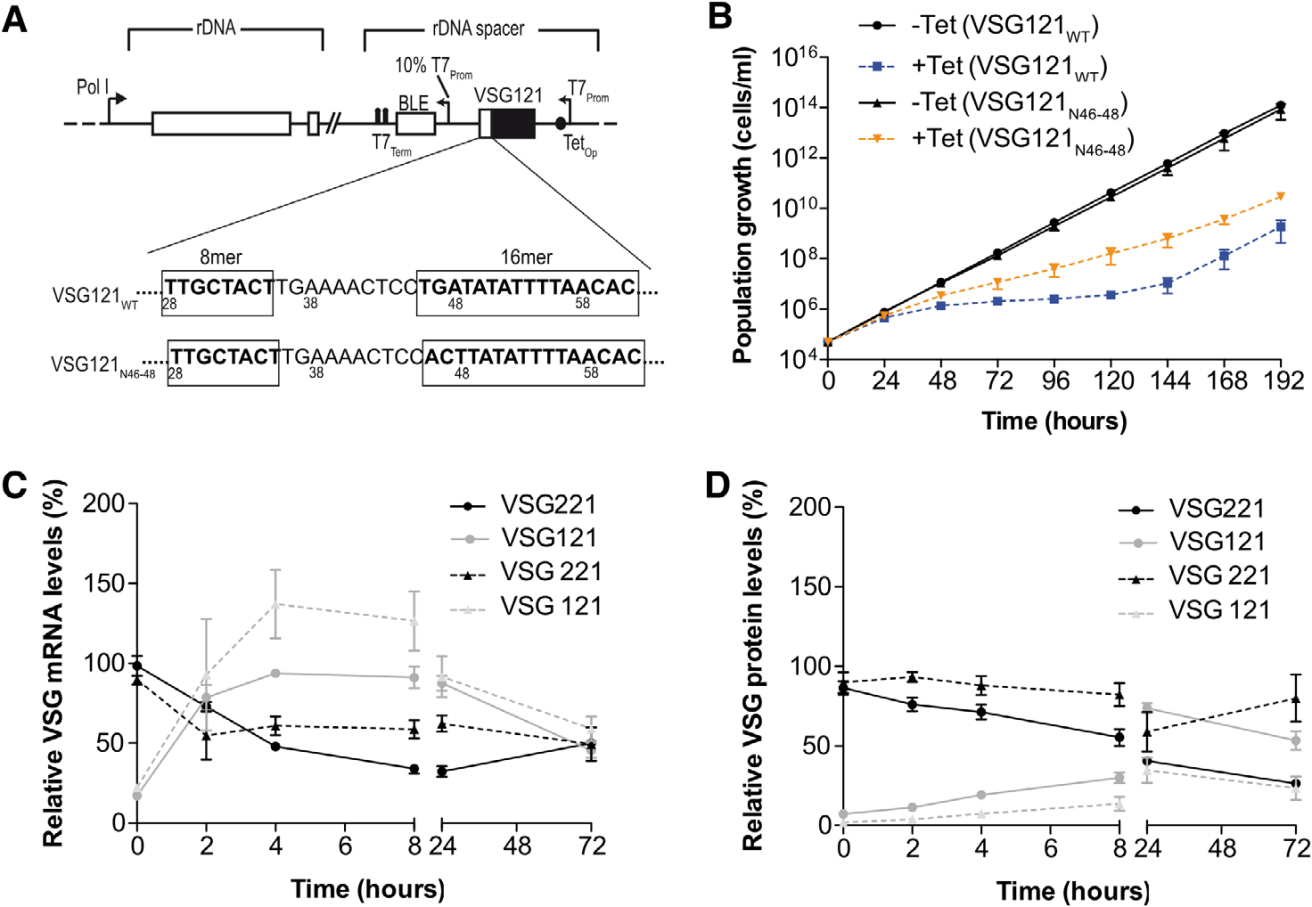
Overexpression of a second VSG with a mutated 16mer motif (VSG121_N46–48_) fails to trigger efficient silencing of endogenous VSG221. (A) Schematic showing the overexpression strategy of the ectopic *VSG121*. (B) Cumulative growth curves of cells before and after induction of VSG121_WT_ and VSG121_N46–48_ overexpression. Three independent clones were analysed for 8 days with or without 1 μg/mL tetracycline. *VSG* mRNA (C) and VSG protein (D) monitored upon induction of VSG121_WT_ and VSG121_N46–48_ overexpression. VSG levels from overexpression of VSG121_WT_ are indicated as circles connected by continuous lines whilst VSG levels from overexpression of VSG121_N46–48_ are indicated as triangles connected by dashed lines. The symbols and lines in black represent the endogenous VSG221 levels whilst the symbols and lines in grey represent the ectopic VSG121 levels. *VSG* mRNA and VSG protein levels were quantified from RNA and protein dot blots, respectively. The *VSG* mRNA was normalised to *tubulin* mRNA and the protein amounts normalised to PFR. The VSG expression levels are given relative to levels in the parental 13–90 (VSG221) or MITat1.6 (VSG121) cells and expressed as the mean ± standard error of the mean (SEM) of three independent clones, respectively.

Since silencing of the VSG located at the expression site occurred independently of the presence of the conserved 16mer in its 3′ UTR, we wondered whether the 16mer motif was critical for expression of the “new” VSG. In our experiment, we mimicked this by inducible overexpression of a second VSG with either a complete 16mer or a mutant version. Would a 16mer 3′ UTR mutant efficiently induce VSG silencing and coat exchange as observed when VSG was overexpressed with wild‐type 3′ UTRs? (Batram et al. 2014; Zimmermann et al. 2017). To test this, we integrated *VSG121* with the mutated 16mer 3′ UTR (VSG121_N46–48_) into a transcriptionally silent rDNA spacer in a parental cell line expressing VSG221. As a control, *VSG121* with an intact 16mer motif (VSG121_WT_) was used (Figure 5A). Overexpression of the ectopic wild‐type VSG121 resulted in the expected severe growth arrest (Figure 5B) (Batram et al. 2014). Induction of the expression of the mutant VSG121_N46–48_, however, caused a much milder growth phenotype. This differential increase in population doubling time may be related to the degree of attenuation of the ES‐resident VSG. Upon overexpression of wild‐type VSG121, the endogenous *VSG221* transcripts decreased by 65% within 24 h, while induction of VSG121_N46–48_ resulted in slower kinetics and a less pronounced decrease of *VSG221* transcripts by about 40%–45% after 24 h. The difference observed cannot be attributed to the degree of VSG overexpression. With VSG121_N46–48_ overexpression, an increase of *VSG121* mRNA to ~90% of wild‐type levels was observed within the first 2 h. There was a further increase to ~120% between 2 and 8 h, levelling to ~90% by 24 h. This was accompanied by a reduction in endogenous *VSG221* transcripts (Figure 5C). The endogenous *VSG221* transcripts however decreased to only 55%–60% within the first 24 h. This is in contrast to the kinetics of silencing observed upon overexpression of the wild‐type VSG121_WT_, where the endogenous *VSG221* transcripts decreased to less than 35% within the first 24 h (Batram et al. 2014). When the VSG121_N46–48_ mutant was overexpressed, the ectopic VSG121 protein reached only about 35% of wild‐type levels after 24 h and decreased to less than 25% by 72 h (Figure 5D). The endogenous VSG221 protein remained abundantly expressed although higher amounts of *VSG121* transcripts were produced. Overexpression of the wild‐type VSG121 on the other hand produced high levels of the ectopic VSG121 protein (~74% by 24 h) and caused a decrease of the endogenous VSG221 protein levels to ~40% by 24 h (Batram et al. 2014). These experiments indicate that overexpression of VSG121_N46–48_ was not effective in suppressing the expression of endogenous VSG221 at both the mRNA and protein levels. Thus, the last two experiments show that an intact 16mer is not required for the VSG gene to be silenced; however, the 16mer may be critical for the newly expressed VSG gene to silence the ES‐resident VSG. These data therefore suggest that 100% conservation of the 16mer is necessary to trigger efficient VSG silencing and coat exchange.

### 2.6 Less Efficient VSG Silencing Is Observed in Slow Growing Cells Upon Overexpression of an Ectopic VSG With a Mutated 16mer Motif

**FIGURE 6:**
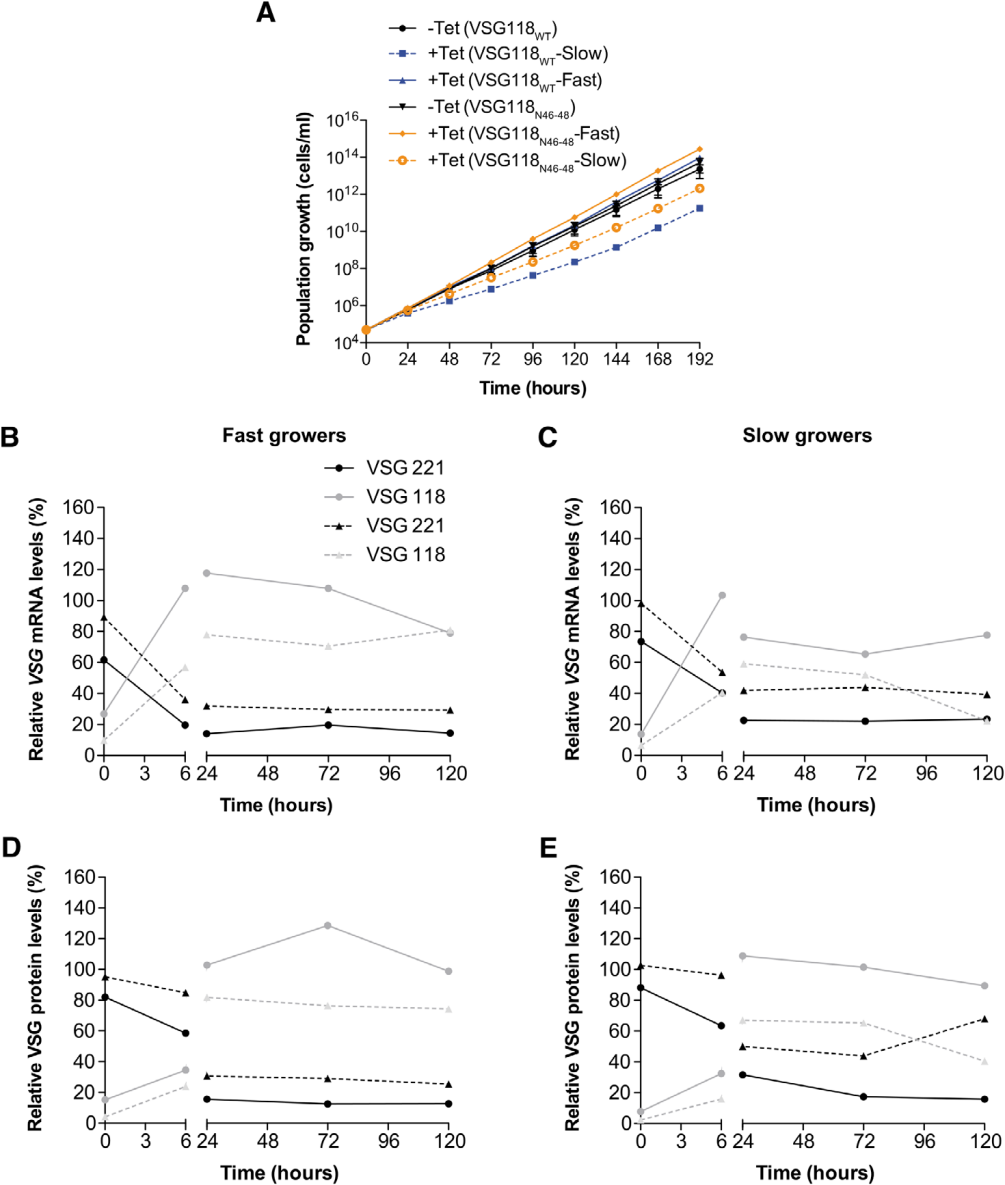
Endogenous VSG silencing is less efficient in slow‐growing cells obtained from VSG118 overexpression. (A) Cumulative growth curves after induction of ectopic VSG118_WT_ and VSG118_N46–48_ overexpression. Two distinct clonal populations (fast growers and slow growers) were observed. The plot for uninduced (‐Tet) cells is from four independent clones and plots for the induced cells (+Tet) are from two clones each. Values are presented as mean. *VSG* mRNA levels in fast growing clones (B) and slow‐growing clones (C) during ectopic overexpression of VSG118_WT_ and VSG118_N46–48_. VSG protein levels in fast growing clones (D) and slow growing clones (E) during ectopic overexpression of VSG118_WT_ and VSG118_N4648_. VSG levels from overexpression of VSG118_WT_ are indicated as circles connected by continuous lines whiles VSG levels from overexpression of VSG118_N46–48_ are indicated as triangles connected by dashed lines. The symbols and lines in black represent the endogenous VSG221 levels whiles the symbols and lines in grey represent the ectopic VSG118 levels. *VSG* mRNA and VSG protein levels were quantified from RNA and protein dot blots, respectively. The *VSG* mRNA was normalised to *tubulin* mRNA and the protein amounts normalised to PFR. Values were expressed relative to levels in the parental 13–90 (VSG221) or MITat1.5 wild‐type cells (VSG118) and are presented as mean.

Our previous work had shown that overexpression of VSG121 causes VSG and ES attenuation, which is followed by dormancy of the parasite (Batram et al. 2014; Zimmermann et al. 2017). This phenotype was particularly striking in the monomorphic MITat 1.2 strain, while in the pleomorphic serodeme AnTat 1.1, *VSG* mRNA was similarly impaired, but ES attenuation and dormancy varied between mutant clones. Thus, we decided to challenge our observations with another VSG; we overexpressed VSG118 with either a wild‐type (VSG118_WT_) or a mutated 16mer (VSG118_N46–48_) from the rDNA spacer. In fact, ectopic overexpression of VSG118_WT_ resulted in two phenotypically different types of clones. One set grew slightly faster than the uninduced cells (fast growers), while the other set grew slower (slow growers). These two growth phenotypes are analoguos to the proliferating and growth‐arrested mutants observed upon VSG118 overexpression in the pleomorphic serodeme AnTat 1.1 (Zimmermann et al. 2017). The slow‐growing cells may therefore represent populations with a partial attenuation of the expression site. VSG silencing and coat exchange occurred with similar kinetics in both slow and fast clones (Figure 6B–E). Overexpression of the mutant VSG118_N46–48_ also produced fast and slow‐growing clones (Figure 6A). In contrast to the overexpression of VSG118 with the wild‐type 3′ UTR (VSG118_WT_), overexpression of the mutant VSG118_N46–48_ caused a much less efficient VSG silencing specifically in the slow‐growing clones (Figure 6B–E). This result supports our finding that an ectopic VSG requires an intact 16mer motif for efficient silencing of the ES‐resident, endogenous VSG.

## 3 Discussion

In this study we demonstrate that the 100% conserved 16mer motif within the VSG 3′ UTR of *T. brucei* plays a role in VSG switching by facilitating efficient silencing and exchange of the active VSG. Our data also confirm the role of the motif in *VSG* transcript stability. Mutating the 16mer reduces the levels of the transcript, consistent with earlier studies (Berberof et al. 1995; Ridewood et al. 2017). However, a tolerated mutation of the motif involving substitution of up to three nucleotides (TGA to ACT) did not lead to loss of cell viability or a failure in VSG protein production. The cells with the mutated 16mer motif grew slower compared to cells with the wild‐type 16mer. Both cell lines however produced high amounts of VSG protein, suggesting that although the 16mer enhances stability to very high levels, the parasite is able to tolerate reduced stability under certain expression configurations. The slower growth of the cells with the mutated 16mer may be due to a reduced rate of protein synthesis as the rate of synthesis of a protein is dependent on the concentration and translation efficiency of its mRNA (Polymenis and Aramayo 2015). Our data therefore showed that the 100% conservation of the 16mer motif is not essential for functional VSG protein production; hence the high conservation of the motif may not solely be due to its role in transcript stability. The caveat to this conclusion is that all of these experiments were carried out in vitro. We do not know if the slow‐growing mutants will be viable under in vivo conditions. The growth rate of these mutants is however similar to the in vitro growth rate of some wild‐type strains of *T. brucei* .

Two mechanisms have been proposed for the maintenance of the stability and abundance of the *VSG* transcripts. One of these mechanisms proposes the presence of a 16mer binding factor which prevents degradation of the mRNA by blocking association of the transcript with nucleases (Ridewood et al. 2017). CFB2 has been identified as the 16mer binding factor and further shown to act by recruiting a stabilising complex that includes MKT1, PBP1, PABP2 and the cap‐binding translation initiation complex EIF4E6/G5 (do Nascimento et al. 2021; Bravo Ruiz et al. 2022). The second proposed mechanism involves a 16mer‐dependent m^6^A modification of poly(A) tails of VSG transcripts which inhibits RNA degradation by blocking deadenylation (Viegas et al. 2022). We found that the presence of the tolerated 16mer mutation did not impact the m^6^A modification of the VSG transcripts, suggesting that the reduction in the transcript level observed is not attributable to impaired m^6^A deposition and that the 100% conservation of the 16mer motif is not required for this modification. This does not however rule out the role the modification plays in transcript stability as subtle differences in m^6^A levels may not be detectable with the experimental approach used.

During a VSG switching event or differentiation of parasites from bloodstream forms to procyclic forms, the active VSG gene is silenced. This occurs due to repression of the active VSG with the expression of a new VSG or procyclin (Myler et al. 1984; Gruszynski et al. 2006). In investigating whether the full conservation of the 16mer is necessary for silencing during differentiation from BSF to PCF, we found that silencing occurs rapidly and efficiently even in the presence of the mutated 16mer. This could mean that silencing during developmental differentiation is primarily driven by transcriptional repression and not destabilisation by a 3′ UTR regulatory element.

For simulation of transcriptional VSG switching, our results interestingly show that when a second VSG is expressed from a non‐ES locus, the intact 16mer in the ectopic VSG 3′ UTR is required for efficient silencing or replacement of the ES‐resident VSG. However, when the mutated 16mer is within the endogenous VSG, silencing of the VSG proceeds in a relatively normal fashion. There is therefore an asymmetry in how much conservation of the 16mer is required depending on whether the mutation is present in the ectopic VSG or the ES‐resident VSG. This asymmetry potentially reflects a mechanistic distinction between silencing and the VSG replacement process. The 16mer probably in addition to stabilising mRNA, influences feedback mechanisms that regulate the activity of the ES or competitive interaction between transcripts for stability‐associated proteins. There is however a caveat that the observed asymmetry may not mirror how transcriptional VSG switching proceeds naturally.

In order to confirm the observation that the intact 16mer in the ectopic VSG 3′ UTR is required for efficient silencing of the ES‐resident VSG, we overexpressed another VSG, VSG118 with either the wild‐type or mutated 16mer. A similar effect was observed although this proceeded differently in the two clonal populations (slow growers and fast growers) upon overexpression of VSG118 with the mutated 16mer. The endogenous VSG221 was not efficiently silenced in the slow growers. Since silencing of the endogenous VSG was impaired upon overexpression of VSG118 with the mutated 16mer only in the slow growers, it suggests that additional factors contributing to ES attenuation may be involved in determining the efficiency of the switch. The overall data however confirm that the 100% conservation of the 16mer motif is important for VSG silencing and coat exchange. The high conservation of the motif appears to be essential in a silent VSG for transcriptional switching between the active VSG and a previously silent VSG. This finding supports the assumption that the mechanism of antigenic switching operates differently in *T. brucei* compared to *T. congolense* and *T. vivax*. Although these other African trypanosome species possess the VSG, they lack the conserved 16mer motif and may therefore have evolved different sequence elements for regulation of stability and transcriptional VSG switching. It must be noted that the simulated in situ VSG switch experiments were carried out by expressing high levels of an ectopic VSG from a non‐ES resident location. Although this directly mimics the process of in situ VSG switching, the natural process is activated from an ES. Some important factors for switching that act directly at the ES of the silent VSG may therefore be missed. We have however made an important contribution to the understanding of the in situ VSG switching process by providing evidence of the involvement of a VSG 3′ UTR specific component, a meticulously conserved 16mer sequence.

Our study has led to the identification of a previously undescribed role of the intact 16mer motif, as the 100% conservation of the motif was found to be essential for triggering efficient VSG silencing and coat exchange during a mimicked in situ VSG switch. This additional role of the 16mer motif in antigenic variation, which is an important process in the parasite, may be the driving force behind the 100% conservation of the motif in all *T. brucei* VSG isoforms.

## 4 Experimental Procedures

### 4.1 Trypanosome Cultivation

All the cell lines generated in this study are based on monomorphic *T. brucei* Lister 427 13–90 cells (Wirtz et al. 1999). Monomorphic *T. brucei* Lister 427 MITat1.6 and *T. brucei* Lister 427 MITat1.5 wild‐type cells were cultivated for use as controls in experiments. The cells were cultivated in HMI‐9 medium (Hirumi and Hirumi 1989) supplemented with 10% heat‐inactivated foetal calf serum (FCS) (Sigma‐Aldrich, St. Louis, USA), at 37°C and 5% CO_2_. Previously transfected plasmids in *T. brucei* 13–90 cells were maintained by the addition of 5 μg/mL hygromycin and 2.5 μg/mL G418. Cell numbers were monitored by counting with a haemocytometer or Z2 Coulter counter (Beckman Coulter). For transfections to generate transgenic cell lines, 3 × 10^7^ *T. brucei* 13–90 cells were electroporated with 10 μg of linearised plasmid DNA using the Amaxa Basic Parasite Nucleofector Kit 1 and Nucleofector II device (programme X‐001) (Lonza, Switzerland).

### 4.2 Plasmids and Generation of Transgenic Trypanosome Cell Lines

For the generation of stable double‐expressor (DEX) cell lines where the ectopic *VSG121* was integrated upstream of the endogenous *VSG221*, the *VSG121* gene with either a wild‐type *VSG121* 3′ UTR or mutated *VSG121* 3′ UTRs was cloned into the plasmid pKD4f and transfected into *T. brucei* 13–90 cells. The intermediate plasmids pBSK.M1.6_WT_ and pBSK.M1.6_∆116–198_ were generated by PCR amplification of *VSG121* from genomic DNA of *T. brucei* MITat1.6 wild‐type cells. The PCR products were digested with NcoI and BamHI and ligated into the plasmid p2084 (MITat1.6T434A in pKD4 vector) digested with the same enzymes to generate the resultant plasmids. For the plasmids pBSK.M1.6_∆63–198_, pBSK.M1.6_∆39–198_ and pBSK.M1.6_∆1–198_, the plasmid pBSK.M1.6_WT_ was used as a template. The PCR products were digested with NcoI and BamHI and ligated into the plasmid p2084 digested with the same enzymes to generate the resultant plasmids. The *VSG121* gene with either a wild‐type or mutated 3′ UTR was subcloned into the pKD4f plasmid after EcoRI digest to yield pES‐M1.6_WT_, pES‐M1.6_∆116–198_, pES‐M1.6_∆63–198_, pES‐M1.6_∆39–198_ and pES‐M1.6_∆1–198_. pBSK.M1.6_∆46–52_ and pBSK.M1.6_∆49–53_ were amplified in two separate PCR steps using the plasmid p2084 as a template. The resulting PCR products were digested with EcoRI and BamHI and ligated into the pBSK plasmid backbone. The PCR products were then excised from the resulting plasmids with SpeI and HincII and subcloned into the pKD4f plasmid to generate pES‐M1.6_∆46–52_ and pES‐M1.6_∆49–53_. For plasmids pBSK.M1.6_Inv28‐35_, pBSK.M1.6_AC41‐42TA_, pBSK.M1.6_C61A_ and pBSK.M1.6_TGA46‐48ACT_, a fusion PCR approach as described by Heckman and Pease (2007) was used to generate the VSG121 3′ UTR mutations. The resulting PCR products were ligated into pBSK plasmid backbones after digestion with EcoRI and BamHI. The *VSG121* CDS with mutated 3′ UTRs were subcloned into pKD4f plasmid after EcoRI digest to yield pES‐M1.6_Inv28‐35_, pES‐M1.6_AC41‐42TA_, pES‐M1.6_C61A_ and pES‐M1.6_TGA46‐48ACT_. The pES plasmids were linearised with AvrII and KpnI and transfected into *T. brucei* 13–90 cells to produce the VSG121 double‐expressor cell lines (WT, ∆116–198, ∆63–198, ∆39–198, ∆1–198, ∆46–52, ∆49–53, Inv28‐35, AC41‐42TA, C61A and TGA46‐48ACT). All plasmids were sequenced to confirm the presence of the desired mutation for the generation of specific mutants.

To generate the VSG121 single‐expressor cell lines ∆221^ES^121_WT_ and ∆221^ES^121_N46–48_, double‐expressor cell lines (221^ES^121_WT_ and 221^ES^121_N46–48_), where the ectopic *VSG121* was integrated downstream of the endogenous *VSG221*, were first generated. The endogenous *VSG221* was then knocked out using the plasmid pJET1.2_M1.2‐Blas_KO. The double‐expressor cell lines 221^ES^121_WT_ and 221^ES^121_N46–48_ were generated by transfecting the plasmids pbRn6.M1.6_WT_ and pbRn6M1.6_TGA46‐48ACT.nPPT_, both linearised with SacI and SalI into *T. brucei* 13–90 cells, respectively. The plasmid pbRn6.M1.6_WT_ was generated as described by (Aroko et al. 2021). pbRn6M1.6_TGA46‐48ACT.nPPT_ on the other hand was generated by mutagenesis PCR using the fusion PCR approach. Briefly, primer pairs C14/HZ35 and C15/HZ32 were used to amplify two PCR fragments from the template pbRn6M1.6_198. The two fragments were joined together in an additional PCR step using the primer pair HZ35/HZ32. The resultant PCR product was cloned into pJET1.2 to yield pJET1.2 M1.6_TGA46‐48ACT_. The M1.6_TGA46‐48ACT_ fragment was then excised with HindIII and MVa1269I and ligated into the pbRn6 backbone obtained from digestion of pbRn6GFP_Δ46–52_ with HindIII and MVa1269I. The upstream integration region of the resultant plasmid was then modified by extending the *VSG221* 3′ UTR sequence to include the native polypyrimidine tract as described for pbRn6.M1.6_WT_.

The cell lines ∆221^ES^121_WT_221^Tet^ and ∆221^ES^121_N46–48_221^Tet^ were generated by transfecting the single‐expressor cell lines ∆221^ES^121_WT_ and ∆221^ES^121_N46–48_ with the overexpression plasmid pRS.221 (a modified pRS121 [Batram et al. 2014] where *VSG121* was replaced with VSG221) after NotI linearisation.

The overexpression cell lines for EP1‐eYFP overexpression (∆221^ES^121_WT_EP1^Tet^ and ∆221^ES^121_N46–48_EP1^Tet^) were generated by transfecting the single‐expressor cell lines ∆221^ES^121_WT_ and ∆221^ES^121_N46–48_ with the overexpression plasmid pRS.EP1::eYFP full3′ UTR. The plasmid was generated by a fusion PCR approach. Briefly, two PCR amplified fragments from the plasmid pGAPRONEΔLII_EP1::eYFP (Engstler and Boshart 2004) were used. Fragment 1 (EP1:eYFP with part of the EP1 3′ UTR, flanked by HindIII and the LII region) was amplified with the primer pair MBS59/MBS60. Fragment 2 (part of the EP1 3′ UTR flanked by XhoI and LII region) was amplified using the primer pair MBS61/MBS62. The two fragments were joined in a PCR using the primer pair MBS59/MBS62. The PCR product obtained (EP1::eYFP_full3′ UTR) was cloned into pJET1.2 to obtain the plasmid pJET1.2_EP1::eYFP_full3′ UTR. The EP1::eYFP_full3′ UTR fragment was then excised from pJET1.2_EP1::eYFP_full3′ UTR using HindIII and XhoI and ligated into the pLEW82v4 vector linearised with the same restriction enzymes. The resultant plasmid pRS.EP1::eYFP_full3′ UTR was linearised with NotI for transfection.

To generate the overexpression cell lines VSG121_WT_ and VSG121_N46–48_, *T. brucei* 13–90 cells were transfected with NotI linearised plasmids pRS.121 (Batram et al. 2014) and pRS.121_N46‐48_, respectively. pRS.121_N46‐48_ was generated using the fusion PCR approach. Two PCR amplified fragments from pbRn6M1.6TGA46‐48ACT.nPPT were joined together. Fragment 1 (VSG121 ORF and part of the 3′ UTR) was amplified using primers MBS71/MBS75. Fragment 2 (part of the 3′ UTR) was amplified using primers MBS72/MBS76. The two fragments were fused together in a PCR using primers MBS71/MBS72. The PCR product obtained (M1.6N46‐48) was cloned into pJET1.2 to obtain the plasmid pJET1.2_M1.6N46‐48. The M1.6N46‐48 fragment was then excised from pJET1.2_M1.6N46‐48 using HindIII and XhoI and ligated into pLEW82v4 vector linearised with the same restriction enzymes.

For the overexpression cell lines VSG118_WT_ and VSG118_N46–48_, *T. brucei* 13–90 cells were transfected with NotI linearised plasmids pRS.118 (a modified pRS.121 plasmid where *VSG221* was replaced with *VSG118*) and pRS.118_N46–48_, respectively. To generate pRS.118_N46–48_, mutagenesis PCR by a fusion PCR approach was used to introduce the mutation TGA46‐48ACT into the VSG118 3′ UTR using the plasmid pRS.118 as a template. Fragments 1 and 2 were amplified using the primer pairs MBS85/MBS86 and MBS87/MBS88, respectively. The two fragments were then fused with primers MBS85/MBS88. The PCR product obtained (M1.5N46‐48) was cloned in pJET1.2 to obtain the plasmid pJET1.2_M1.5N46‐48. The M1.6N46‐48 fragment was then excised from pJET1.2_M1.5N46‐48 using HindIII and XhoI and ligated into the pLEW82v4 vector linearised with the same restriction enzymes.

### 4.3 Differentiation of Monomorphic Bloodstream Trypanosomes to the Procyclic Form

Differentiation of monomorphic bloodstream trypanosomes to the procyclic form was carried out in vitro. BSF trypanosomes were grown to a density of 1.5 × 10^6^ cells/ml in HMI‐9 medium. 2.5 × 10^7^ cells were harvested by centrifugation (1400× *g* for 10 min at room temperature (RT)) and resuspended in 5 mL of Differentiating Trypanosome Medium (DTM) containing 6 mM *cis*‐aconitate (Overath et al. 1986). The parasites were then cultivated at 27°C and 5% CO_2_. The cells were diluted with SDM‐79 medium supplemented with 10% heat‐inactivated FCS when the cell density was above 2 × 10^7^ cells/mL.

### 4.4 RNA Isolation and mRNA Quantification

Total RNA was extracted from 1 × 10^8^ trypanosome cells using the Qiagen RNeasy Mini Kit (Qiagen, Netherlands) following the manufacturer's instructions. Poly(A) + mRNA on the other hand was isolated from total RNA using the Oligotex mRNA Mini Kit (Qiagen, Netherlands) following the manufacturer's protocol. *VSG* mRNA levels were quantified using RNA dot blots as described (Batram et al. 2014).

### 4.5 N6‐Methyladenosine Immunoblotting

Immunoblotting to detect m^6^A modification was carried out as described by Viegas et al. (2022) with slight modifications. DNase I‐treated total RNA (2 μg) or poly(A) + mRNA (50 ng) samples were mixed with a 5× RNA loading buffer (30.8% (v/v) formamide, 2.6% (v/v) formaldehyde, 20% (v/v) glycerol, 0.2% (w/v) bromophenol blue, 4 mM EDTA pH 8) in a ratio of 1 volume 5× loading buffer to 4 volumes of RNA sample. Samples were denatured by heating (70°C for 5 min) and immediately transferred onto ice. The samples were then loaded and resolved on a 1.4% denaturing agarose gel (1.4% agarose, 6.3% formaldehyde, 1× MOPS buffer) at 100 V until the samples had migrated halfway across the gel. The RNA was transferred overnight to a Hybond‐*N*+ nylon membrane (GE Healthcare) by upward capillary transfer with 20× SSC buffer. The RNA was then UV‐crosslinked to the membrane (Stratalinker 2400, autocrosslink: 120 mJ/cm^2^). Membranes were stained with 0.02% methylene blue diluted in 0.3 M sodium acetate (pH 5.5) for 5 min and washed in RNase‐free water. After imaging, the methylene blue was removed by incubation in destaining solution (0.2× SSC, 1% SDS) and washed 3 times in PBST (PBS pH 7.4 with 0.2% Tween‐20). The membranes were then blocked by incubation in 5% skimmed milk in PBST for 1 h followed by overnight incubation with 1 μg/mL mouse anti‐m^6^A antibody (1:1000 in 2.5% skimmed milk in PBST) at 4°C. Membranes were washed three times in PBST and then incubated with HRP‐conjugated anti‐mouse IgG diluted 1:10,000 in 2.5% skimmed milk in PBST for 1 h at room temperature. Membranes were again washed three times in PBST (10 min each) and the signal developed using a Western Lightning Plus‐ECL, Enhanced Chemiluminescence Substrate kit (PerkinElmer). Images were acquired using the iBright FL1000 imaging system (Thermo Fisher Scientific).

### 4.6 Protein Analysis

Protein amounts were quantified using either western blots or protein dot blots. For western blots protein samples from 1 × 10^6^ trypanosome cells were separated on 12.5% sodium dodecyl sulphate (SDS)‐polyacrylamide gels and transferred onto nitrocellulose membranes (GE Healthcare Life Sciences). For protein dot blots, 3 μL of protein samples containing 6 × 10^5^ cell equivalents were loaded onto nitrocellulose membranes fixed in a dot blotter. The membranes were blocked by incubation with 5% milk powder in PBS for 1 h at room temperature (RT) or overnight at 4°C. Primary antibodies (rabbit anti‐VSG221; 1:5000, rabbit anti‐VSG121; 1:2000, rabbit anti‐VSG118; 1:10,000 and mouse anti‐PFR antibody (L13D6, 1:20) (Kohl et al. 1999)) were then applied in PBS/1% milk/0.1% Tween‐20 solution for 1 h at RT. After four washes (5 min each) with PBS/0.2% Tween‐20, IRDye 800CW‐conjugated goat‐anti‐rabbit and IRDye 680LT‐conjugated goat‐anti‐mouse secondary antibodies (1:10,000; LI‐COR Biosciences) were applied in PBS/1% milk/0.1% Tween‐20 solution for 1 h at RT in the dark. The membranes were washed four times (5 min each in the dark) with PBS/0.2% Tween‐20 followed by a final 5 min wash with PBS. Blots were analysed using a LI‐COR Odyssey system (LI‐COR Biosciences).

## Author Contributions

**Majeed Bakari‐Soale:** investigation, writing – original draft, methodology, visualization, writing – review and editing. **Christopher Batram:** investigation, methodology, validation. **Henriette Zimmermann:** investigation, validation, methodology. **Nicola G. Jones:** conceptualization, writing – original draft, methodology, validation, supervision. **Markus Engstler:** conceptualization, investigation, funding acquisition, writing – original draft, methodology, validation, visualization, writing – review and editing, project administration, resources, supervision.

## Supporting information


**Figure S1:** mmi70031‐sup‐0001‐AppendixS1.pdf.

## Data Availability

The data that support the findings of this study are openly available in bioRxiv at https://www.biorxiv.org, reference number https://doi.org/10.1101/2023.12.21.572740.
