# Supplementary material for: The Dual Role of the 16mer Motif Within the 3′ Untranslated Region of the Variant Surface Glycoprotein of Trypanosoma brucei
Source: Mol Microbiol. 2025 Nov 17;125(1):67–79. doi: 10.1111/mmi.70031 (PMC12763537; doi:10.1111/mmi.70031)
Supplement: Supplementary file 1 — Figure S1: mmi70031‐sup‐0001‐AppendixS1.pdf. [file MMI-125-67-s001.pdf]

Supporting information

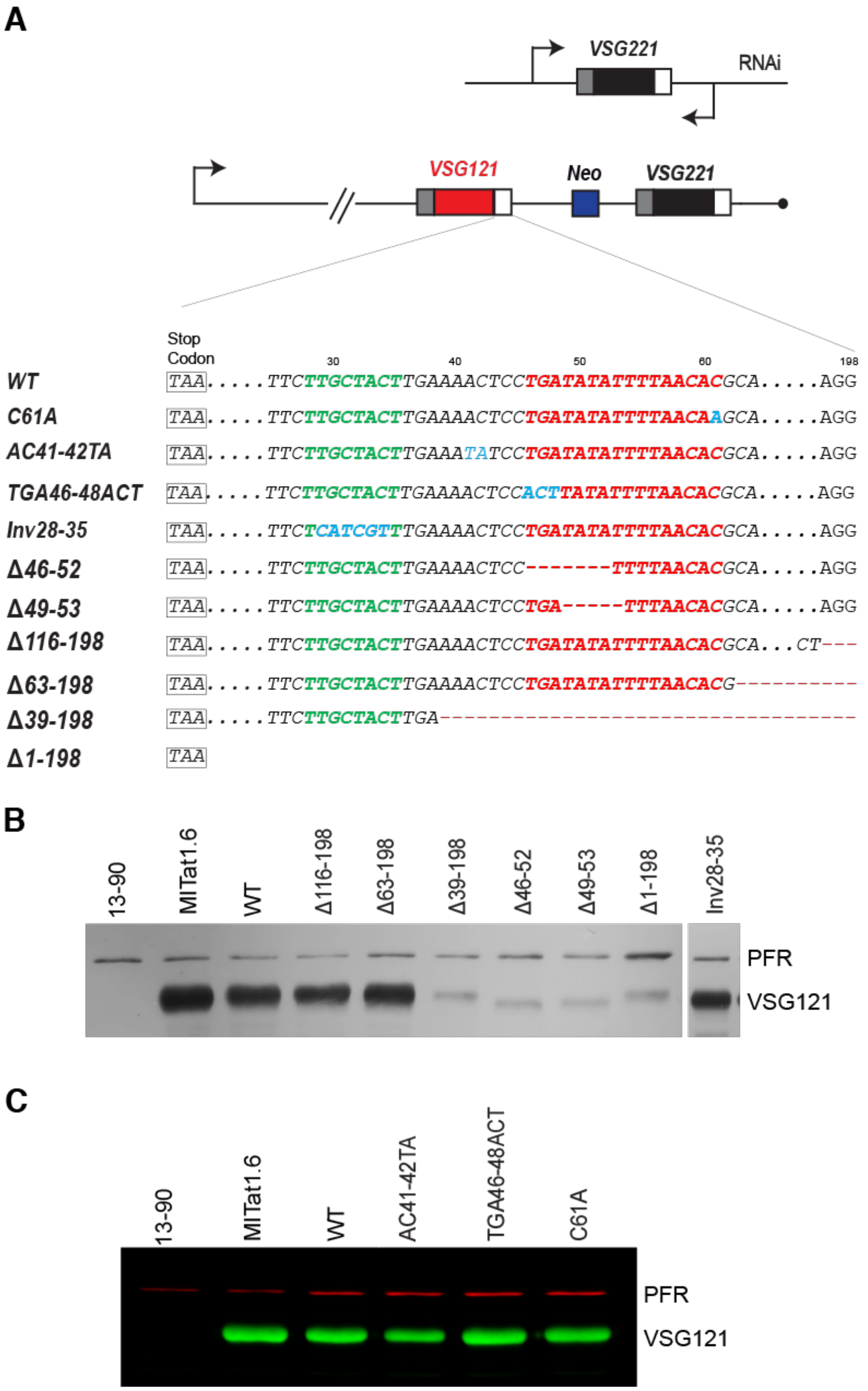

Figure S1. Mutational analysis of the VSG121 3´ UTR. (A) Schematic of the inducible VSG221 RNAi double-expressor cell line with specific mutations of the VSG121 3´ UTR. NEO: Neomycin

resistance cassette. Nucleotides in green represent the 8mer motif, red nucleotides represent the 16mer motif and the blue nucleotides are substituted nucleotides within the 3′ UTR. Dashes represent deleted nucleotides and dots are extensions of the sequence. (B, C) Western blots showing VSG121 protein levels upon mutations in the VSG121 3′ UTR. Note that the 3′ UTR mutants Δ46-52 and Δ49-53 were tested using an N-glycosylation deletion mutant of VSG 121 (Hartel et al., 2016). This explains the slightly reduced size of the protein.

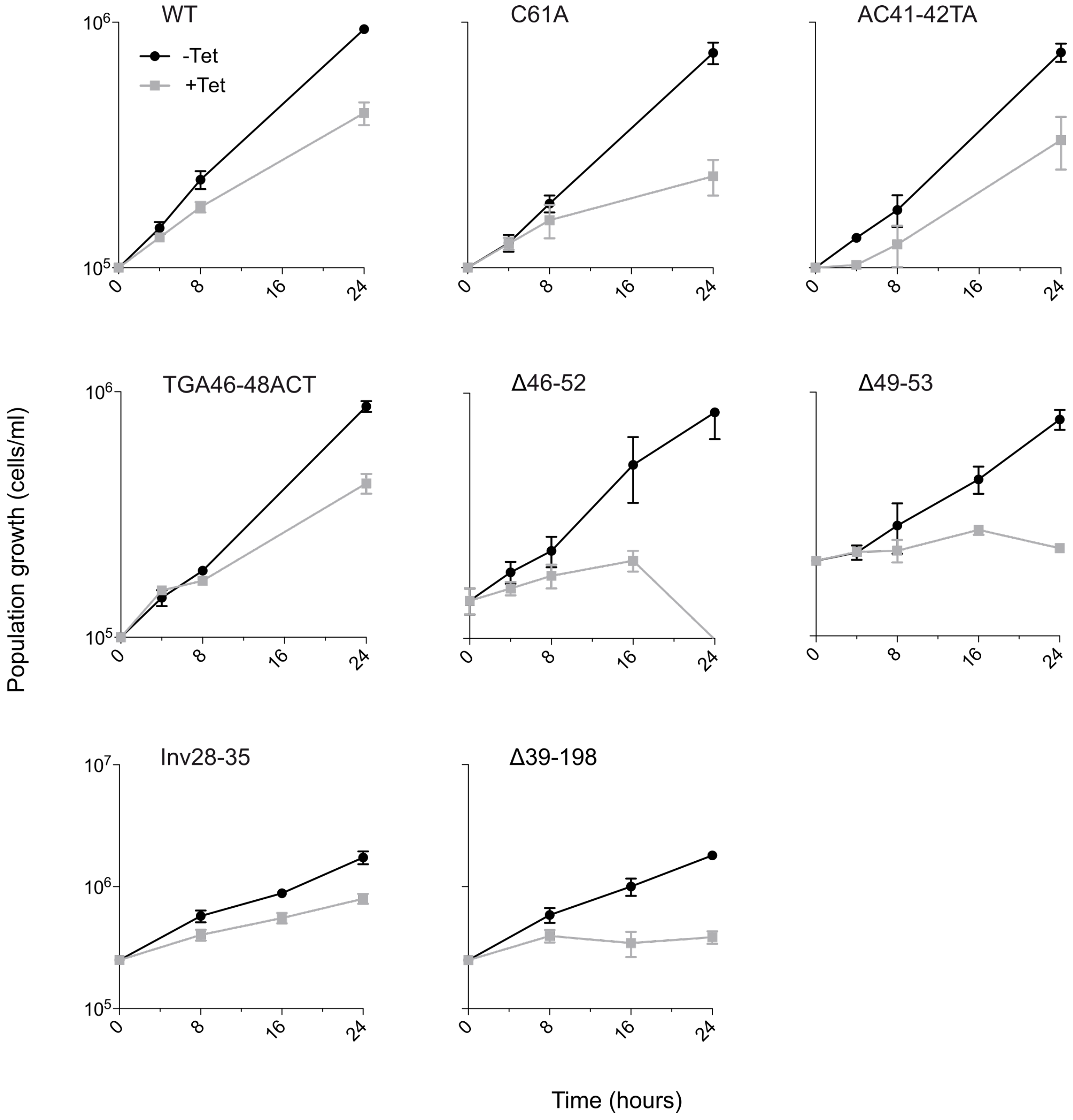

Figure S2. VSGs with tolerated mutations support cell growth upon RNAi-mediated depletion of VSG221. Cumulative growth curves of double-expressor cells upon RNAi-mediated depletion of VSG221. Mutations Δ46-52, Δ49-53 and Δ39-198 are not tolerated. They do not support growth as can be seen in the cell density at 24h compared to the Wt and other mutants.

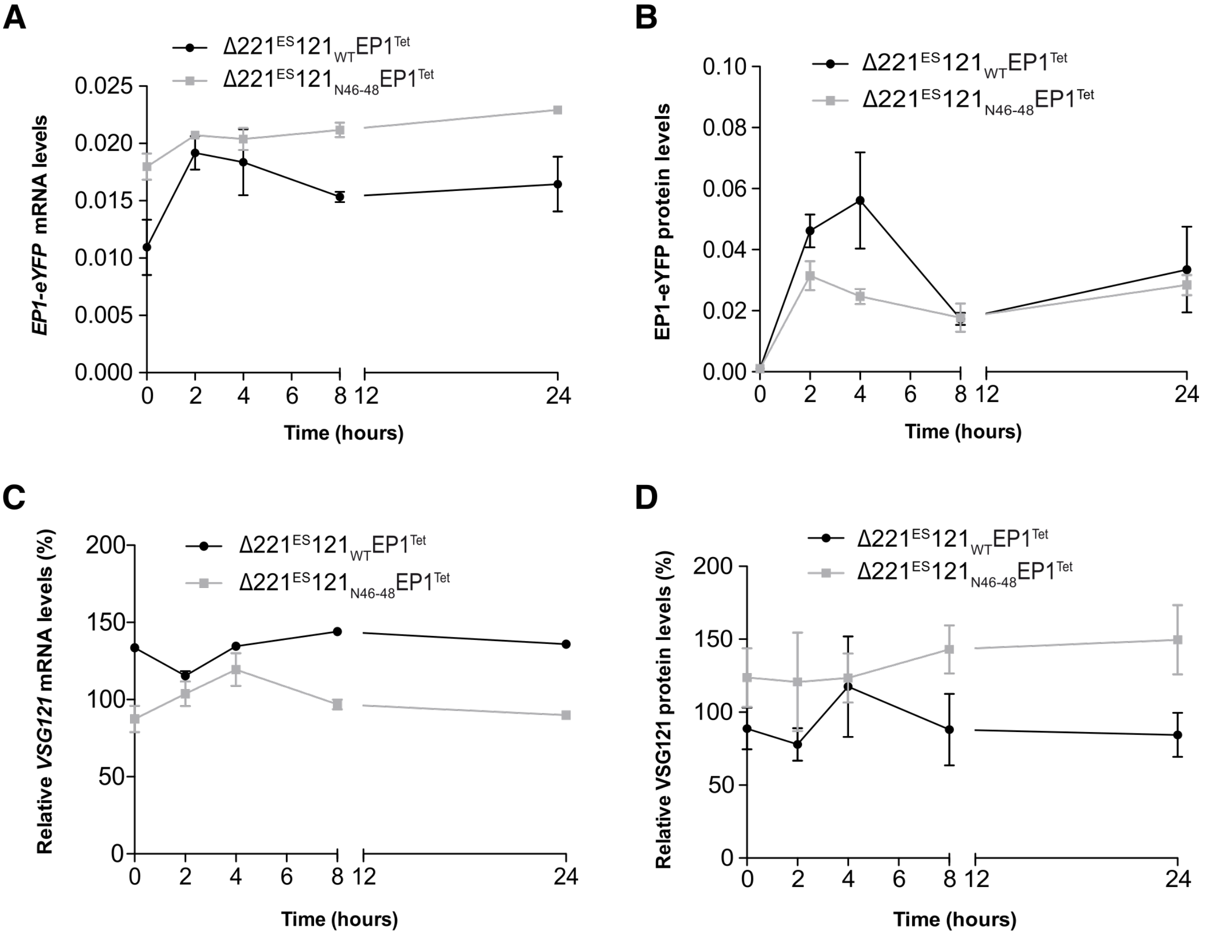

Figure S3. Overexpression of EP1-eYFP in $\Delta221^{ES}121_{WT}$ and $\Delta221^{ES}121_{N46-48}$ single-expresser cell lines. *EP1-eYFP* mRNA (A) and EP1-eYFP protein (B) monitored in $\Delta221^{ES}121_{WT}EP1^{Tet}$ and $\Delta221^{ES}121_{N46-48}EP1^{Tet}$ cells during the course of EP1-eYFP overexpression. *EP1-eYFP* mRNA and EP1-eYFP protein were normalised to *tubulin* mRNA and PFR, respectively. Values are expressed as mean ± standard error of the mean (SEM) of three clonal cells each. *VSG121* mRNA (C) and VSG121 protein (D) monitored in $\Delta221^{ES}121_{WT}EP1^{Tet}$ and $\Delta221^{ES}121_{N46-48}EP1^{Tet}$ cells during the course of EP1-eYFP overexpression. *VSG* mRNA and VSG protein levels were quantified from RNA and protein dot blots, respectively. *VSG* mRNA was normalised to *tubulin* mRNA and the protein amounts normalised to PFR. The VSG expression levels are given relative to levels in the parental MITat1.6 (VSG121) cells and expressed as mean ± standard error of the mean (SEM) of three clonal cell lines, respectively.

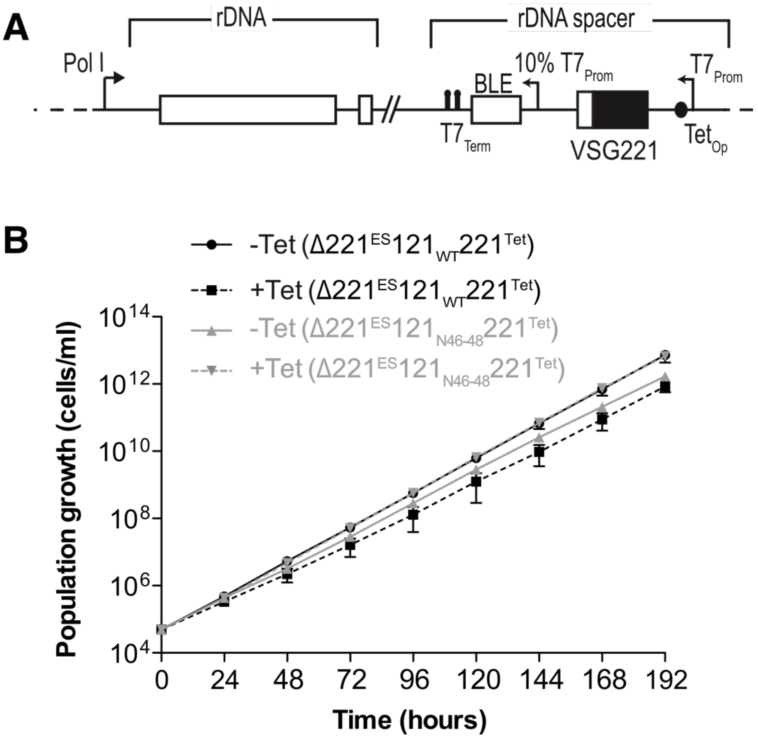

Figure S4. Overexpression of VSG221 in $\Delta 221^{ES}121_{WT}$ and $\Delta 221^{ES}121_{N46\text{-}48}$ single-expresser cell lines. (A) Schematic of the ectopic overexpression system (adapted from Batram et al., 2014). Cumulative growth curves of $\Delta 221^{ES}121_{WT}221^{Tet}$ (black) and $\Delta 221^{ES}121_{N46\text{-}48}221^{Tet}$ (grey) cell lines over the course of VSG221 overexpression.
